# 2474 Promoting collaboration among researchers: A team science training curriculum

**DOI:** 10.1017/cts.2018.222

**Published:** 2018-11-21

**Authors:** Jacqueline Knapke, Amy Short, Tamilyn Bakas, Jacinda Dariotis, Elizabeth Heubi, Saundra Regan, Barbara Speer, John Kues

**Affiliations:** University of Cincinnati

## Abstract

OBJECTIVES/SPECIFIC AIMS: As multidisciplinary, interdisciplinary, and transdisciplinary research has become imperative to solving the complex problems of contemporary healthcare, teaching researchers how to create and maintain high-functioning and innovative teams has also become paramount. In Fall 2016, the Center for Improvement Science (CIS) core, in collaboration with the Translational Workforce Development (TWD) core, at the Cincinnati Center for Clinical & Translational Science & Training (CCTST) began offering training in Team Science in an effort to better prepare researchers for collaborative work. Since then, the CIS has expanded Team Science education into a multifaceted and adaptable curriculum that includes workshops, team consultations, Grand Rounds, grant writing assistance, grant review, train-the-trainer, and a graduate-level course. METHODS/STUDY POPULATION: Over almost 2 years, we have offered 9 unique workshops attended by individuals from the University of Cincinnati, UCHealth, and Cincinnati Children’s Hospital Medical Center. Recruitment was primarily accomplished via email invitations. Topics ranged from introductory team science issues such as Creating Teams, Team Effectiveness, and Team Leadership to more advanced team science areas such as Team Dysfunctions and Conflict Management. In addition, we have consulted with researchers on Team Science components of grant applications and served as grant reviewers for Team Science elements in a competitive, internal research funding program. We have developed tools and teaching strategies for faculty members tasked with teaching students about collaboration (train-the-trainer). And finally, we offered a graduate level course on Collaboration and Team Science. RESULTS/ANTICIPATED RESULTS: Over 250 participants attended our workshops and Grand Rounds, many at the faculty level, but we also had research staff and graduate students register. Content was very well-received, with workshop evaluations typically scoring in the high 4.5 and above range (on a 5-point scale, with 5 being the highest rating). The CIS team received (and accepted) at least 2 follow-up invitations from workshop participants to provide training to an additional team or group. We are tracking data on long-term effects of team science training and consultation, both in research productivity and team satisfaction/longevity. DISCUSSION/SIGNIFICANCE OF IMPACT: The goals of Team Science training at the Cincinnati CCTST are 2-fold: to provide practical knowledge, skills, and tools to enhance transdisciplinary collaboration and to promote systemic changes at UC, CCHMC, and UCHealth that support team science. After almost 2 years of training, team science is gaining traction among key leaders at our local institutions and a broader audience of researchers who see how collaborative practice can enhance their professions.

